# Microtechnology-Based Multi-Organ Models

**DOI:** 10.3390/bioengineering4020046

**Published:** 2017-05-21

**Authors:** Seung Hwan Lee, Jong Hwan Sung

**Affiliations:** 1 School of Chemical and Biological Engineering, Seoul National University, Seoul 151-742, Korea; skulsh78@snu.ac.kr; 2 Department of Chemical Engineering, Hongik University, Seoul 121-791, Korea

**Keywords:** microtechnology, in vitro models, multi-organ chip, multiple organ interaction, microfluidics

## Abstract

Drugs affect the human body through absorption, distribution, metabolism, and elimination (ADME) processes. Due to their importance, the ADME processes need to be studied to determine the efficacy and side effects of drugs. Various in vitro model systems have been developed and used to realize the ADME processes. However, conventional model systems have failed to simulate the ADME processes because they are different from in vivo, which has resulted in a high attrition rate of drugs and a decrease in the productivity of new drug development. Recently, a microtechnology-based in vitro system called “organ-on-a-chip” has been gaining attention, with more realistic cell behavior and physiological reactions, capable of better simulating the in vivo environment. Furthermore, multi-organ-on-a-chip models that can provide information on the interaction between the organs have been developed. The ultimate goal is the development of a “body-on-a-chip”, which can act as a whole body model. In this review, we introduce and summarize the current progress in the development of multi-organ models as a foundation for the development of body-on-a-chip.

## 1. Introduction

Drugs are characterized by their pharmacokinetic (PK) and pharmacodynamic (PD) profiles [1,2,3]. The ability to accurately predict the PK-PD profile in the early stages of drug development can reduce the failure rate of the development process [4]. However, as numerous parameters affect the complex ADME processes, the prediction of the efficacy of a drug on the body is difficult [5]. Inaccurate predictions can result in toxicity or low efficacy, which is a major factor in the failure of new drug candidates [6]. As the failure of new drugs leads to the reduction of investment, an accurate evaluation of the drug during the initial stages of development is essential [7]. In order to aid the drug development process, mathematical model-based PK-PD profiling has been developed and used [8]. The modeling processes require information on the PK profile and large quantities of PD data [4]. However, the unavailability of human clinical and animal research data in the initial stage of new drug development, owing to economic and ethical concerns, causes a shortage of information on the drug effect-related parameters [2]. PK-PD models were developed to overcome these issues [9], but because conventional cell culture models are used for PK-PD models, the predictions still widely differ from the in vivo results [9]. Thus, improved in vitro prediction models are required for an accurate prediction of the efficacy of a drug following administration into the body [2].

Microtechnology is a scientific field for manipulating and manufacturing fluids with structures of micrometer scales and is currently widely applied to various research fields, including chemistry and biology [10]. Compared to the traditional cell-based analyses, microtechnology allows a highly efficient analysis of reactions with a low consumption of specimens, low cost of reagents, and fast reaction time [11]. In addition, the cell chip technology can be implemented by combining it with cell culture technology [12]. Microtechnology provides an opportunity for duplicating the microstructure and environment of in vivo systems and, thereby, a small chip can be used to study the cell-cell and cell-extracellular matrix (ECM) interactions [13,14]. Moreover, because the components representing the organs can be connected through the microfluidic channels, simulating the interactions between multiple organs becomes feasible, which implies that the PK-PD model can be physically implemented [15]. In addition, the action of drugs in the body can widely vary, depending on the mode of administration. Various chip systems have been developed to recapitulate different modes of drug administration, mainly focusing on two major modes of drug delivery, oral administration and bolus injection [16].

In this review, we summarized PK-PD models to improve the toxicity and efficacy analysis of drugs. The microtechnology-based multiple organ model for monitoring the inter-organ interactions is highlighted. First, we introduce gut-liver interaction-based first pass metabolism. We describe a system that simulates first pass metabolism based on the cell line, co-culture with organ-specific cells, primary cell, and tissue slice. Then, more than three organ system-based models and PK-PD model-based systems are summarized. Finally, we discuss and suggest future guidance for the development of a general body model.

## 2. Pharmacokinetic-Pharmacodynamic (PK-PD) Modeling

### 2.1. PK Modeling

Here, we briefly describe the concept and methodology of PK modeling, with a specific emphasis on PBPK (physiologically-based pharmacokinetic) modeling. The main purpose of this section is to introduce to readers the concept of PK and PBPK modeling, and describe how this modeling approach can be useful for designing and analyzing the multi-organ chip systems, which will be introduced later. For a more complete review on the concept and applications of PK models, there are several comprehensive reviews on this subject [17,18,19,20]. PK refers to the study and quantification of ADME processes [4,21]. PK models, which are mathematical models that predict the drug concentration and time at a given dose, can be categorized into empirical and mechanistic models, according to how they are constructed. One- or two- compartment PK models assume that the drug concentration quickly reaches equilibrium after administration into the body. It is assumed that administered drugs are quickly distributed throughout the body, and reach equilibrium within the apparent body volume. A two-compartment PK model generally consists of a central compartment and a peripheral compartment. The central compartment assumes that the sum of the drugs that are administered is distributed uniformly in all organs and the peripheral compartments contain the drugs remaining in the less-well perfused organs. By solving the mass balance equations, two-compartment PK models are mathematically represented by the sum of the two exponential terms of biphasic straight lines in a log scale plot [22].

**Figure 1:**
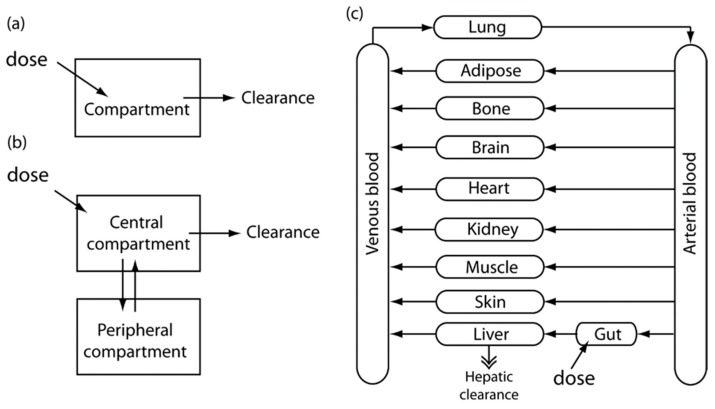
Schematic diagrams for the basic concept of PK models. Image reprinted from [4] with the permission of John Wiley & Sons. (**a**) One-compartment model; (**b**) Two-compartment model; (**c**) A more complex PBPK model.

Physiologically-based pharmacokinetics (PBPK) present a mechanistic model. PBPK models integrate actual physiological parameters, such as the anatomical structure of model organism-based organ size or blood-flow velocity. The inflow, outflow, and reaction of drugs in each compartment are explained by mass balances, the set of which can be numerically solved for the entire system. Although physiological parameters are easily found in the literature, the enzyme kinetics or partition coefficients (PCs) between tissue and blood cannot be easily determined. The optimization of parameters was performed by fitting a model to experimental data based on the least squares methods and the Bayesian technique [23]. Figure 1 shows schematic diagrams showing the basic concept of PK models, starting from a simple one-compartment and two-compartment models, and advancing to a more complex PBPK model.

### 2.2. PD Modeling

PD describes the time-dependent effects that occur in response to drugs; these effects are based on the interaction between the drug and the body. Therefore, a PD model can be formed based on the drug mechanism. PD modeling can be initiated by the expression of drug effects by using a drug concentration based on a linear or non-linear function, which should describe the action of the drug. In some drugs, a linear relationship is used, i.e., the pharmacological effect of a drug is directly proportional to the concentration of the drug in the body. For example, the following equation can be used to describe the linear relationship of the drug’s effect to the dose:

(1) $$E=E_{0}+S\times C$$

The *E_max_* model, which is commonly used, defines a concentration value for the maximal effect of a drug [24]. Although the concentration of the drug may increase beyond this value, the corresponding pharmacological effect is not enhanced. For example, the following equation can be used to describe the action of drugs based on the *E_max_* model:

(2) $$E=\frac{E_{max}\times C}{EC_{50}+C}+E_{0}$$

Such PD models cannot accurately describe the time delays or irreversible effects that are detected with many drugs, because they assume that the body’s physiological responses to drug administration are immediate and reversible, which is sometimes not the case. For example, some chemotherapeutic drugs can cause irreversible cell death a considerable time after drug administration [25]. This indicates that time-dependent effects should be considered by establishing the change in a physiological response as a function of drug concentration, and irreversible effects should be incorporated into the models.

A combination of the PBPK and PD models allows the prediction of the pharmacological effects on a target site or organ from a given dose. The combined PK-PD model could be considered a dynamic model because it includes a time-dependent variable. Therefore, a PK-PD model should be able to predict the outcome based on the initial drug dose [3,26]. While PK-PD models are purely mathematical, they can be more powerful once they are combined with other physiological models, either in vivo or in vitro models. In the following section, we introduce and summarize the concept of multi-organ models and illustrate how these systems can function as physical models of the interactions between multiple organs.

## 3. Two-Organ-Based Organ Models

Mimicking the intestine and the liver is essential for reproducing the first pass metabolism of drugs. An orally administered drug goes through the intestine and the liver before reaching the systemic circulation. Connected co-culture of the intestine and the liver has been attempted. The chamber simulating the two organs was integrated into a microchip and connected by a fluid network [27,28]. Thereby, a simulated first pass metabolism system was constituted, which became a representative model for monitoring the interaction between organs [15]. In first pass metabolism, food and drugs are absorbed along the small intestine, delivered to the liver through the blood circulation, and metabolized in the liver by phase I and phase II metabolism [29]. Therefore, most systems are designed for analyzing metabolites in the small intestine, which are formed in the first chamber and subsequently forwarded to the liver, where they are further metabolized (as simulated in the second chamber) [30]. This design enables the study of the effect of the metabolites or the medium of the intestines on the metabolism in the liver. In addition, it enables the study of the first pass metabolism in other tissues by adding another chamber to the microfluidic system [31]. 

### 3.1. Cell Line-Based Two-Organ Models

Several researchers have employed cell lines in simulating first pass metabolism. Cell lines are easier to handle than animals or primary cells. Furthermore, cell lines are cost-effective tools because they can proliferate indefinitely under established conditions. For mimicking the intestine, the Caco-2 cell line that originates from colon cancer has been widely used, and it has shown several key features such as the barrier function and expression of transporter-related genes. For mimicking the liver, the HepG2 cell line has been used in many studies. The HepG2 cell line is derived from the human hepatocellular carcinoma cell and has been applied to drug metabolism and toxicity studies of the liver. One of the predominant examples reported the comparison between the dynamic co-culture and static co-culture, and the monoculture system. The Leclerc group [32] used a bioreactor system that consisted of a polycarbonate cell microfluidic platform and cell culture insert. The Caco-2 cells were cultured in the cell culture insert for mimicking the apical and basolateral compartments of the intestine. The basolateral compartment was connected with the microfluidic compartment where HepG2 cells were cultured. The outlet of the microfluidic part was connected to another well for sampling metabolites. The flow was controlled using a peristaltic pump so that a perfusion-based dynamic co-culture of Caco-2 and HepG2 cells could be implemented. The viability, integrity, and functionality of the Caco-2 cells, as well as the viability and metabolism of the HepG2 cells, were maintained in the system. In addition, the first pass metabolism was verified with an observation that phenacetin was metabolized to acetaminophen by the CYP1A enzyme in the liver cells. The production of acetaminophen and the biotransformation rate of phenacetin in the perfusion-based dynamic co-culture system were higher than that observed using the static co-culture and monoculture systems, implying the superiority of the perfusion culture system. 

Several studies have observed that the metabolite of first pass metabolism could affect other cells. One example from Kimura et al. [33] describes an on-chip small intestine–liver-coupled model for pharmacokinetic studies. The microfluidic system consisted of the small intestine, liver, and lung chambers. A stirrer-based micropump was also integrated into the system. To simulate the apical and basolateral sides of the intestine, the small intestine compartment was separated by the microporous polyethylene terephthalate membrane. The polyethylene terephthalate membrane has a low pore density and high transparency [34]. Caco-2, HepG2, and A549 cells were cultured on the chip for use as an organ model for the small intestine, liver, and lung, respectively. By using a microfluidic system, the structure of internal circulation, the volume ratios of each organ, and the blood flow ratio of the portal vein to the hepatic artery were mimicked. The blood flow ratio of the portal vein to the hepatic artery in the human body was maintained at 3:1 by calculating the pressure drop. The effects of epirubicine (EPI), irinotecan (CPT-11), and cyclophosphamide (CPA) on the target cells were demonstrated using this system. Because the Caco-2 cell monolayer functions as a barrier against hazardous chemicals, the viability of HepG2 cells against EPI increased when the Caco-2 cell was co-cultured on the chip. CPT-11 and CPA were metabolized by HepG2 cells and showed anticancer effects for the viability of A549 cells.

The Shuler group [35] designed a microfluidic system, termed the microscale cell culture analog (μCCA), to mimic the intestine and the liver. The gastrointestinal (GI) μCCA was fabricated as a model of the intestine. The Caco-2 cells formed layers on a microporous polycarbonate membrane that separated the system into apical and basal parts. The membrane used was a commercially available, semi-permeable membrane commonly used for cell culture. The multiple chamber-based μCCA was fabricated by conventional microfabrication technology. The multiple chamber-based μCCA consisted of the liver, lungs, and other tissue compartments. Each compartment was connected by a microchannel. GI μCCA and multiple chamber-based μCCA were connected to simulate the absorption of acetaminophen and metabolism. Acetaminophen was transferred to the liver (HepG2) through the Caco-2 cell layer. After a metabolic reaction, it was observed that acetaminophen affected the lungs (L2). The L2 cells were damaged by the metabolite of acetaminophen. As the lung cells have a lower amount of phase II enzymes than the liver cells, the viability of the lung cells was lower than that of the liver cells. These studies showed that the gut–liver interaction for mimicking first pass metabolism can be reproduced on a single chip system. Furthermore, these systems can be applied to the efficacy of drugs that are taken orally for other organ/tissues and cancer cells.

### 3.2. Co-Culture of Organ Specific Cells

**Figure 2:**
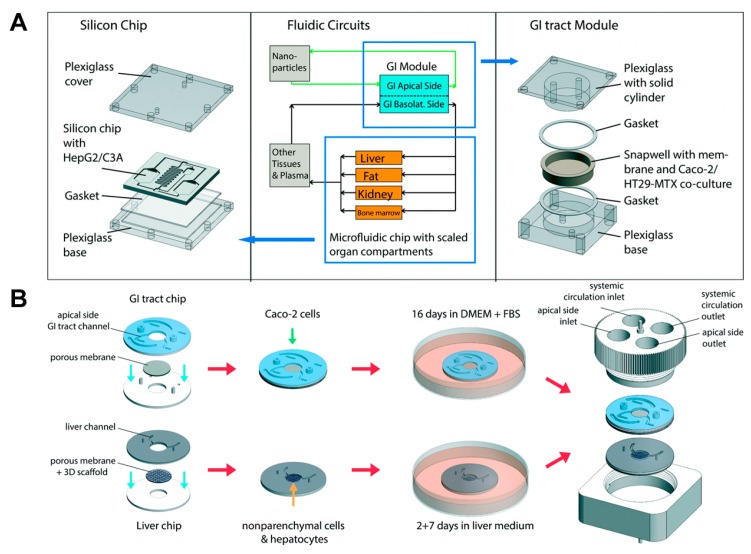
First pass metabolism models based on co-culture with organ-specific cells. (**A**) The GI tract module and the body-on-a-chip system where Caco-2 and HT29-MTX cells were co-cultured and connected with HepG2 cells. Image reprinted from [31] with the permission of The Royal Society of Chemistry. (**B**) Modular, pumpless body-on-a-chip system where Caco-2 cells were cultured on the GI tract chip, and hepatocyte and non-parenchymal cells were co-cultured on the liver chip, respectively. Then, each module was connected to simulate first pass metabolism. Image reprinted from [39] with the permission of The Royal Society of Chemistry.

Several studies have reported that Caco-2 cells can be co-cultured with other organ-specific cells such as goblet cells and lymphocytes. It is known that the co-culture of Caco-2 cells and other cells allows for an improvement of the gut function. The Shuler group integrated mucous secreting cells (HT29-MTX) to the GI μCCA to simulate in vivo cell composition [29]. Caco-2 and HT29-MTX cells are representative cell types of the small intestine, and the co-culture of these cells allows for the formation of a tight junction and mucus layer [36,37,38]. Moreover, chyme was added to the system to achieve an absorption condition similar to the in vivo absorption in the small intestines. Acetaminophen passed through Caco-2/HT29-MTX cells in the GI μCCA and decreased glutathione levels. Then, acetaminophen was transported to the liver sector, which led to liver cell death because the metabolism of acetaminophen caused a decrease in glutathione. The toxicity of acetaminophen was examined, and the metabolism of acetaminophen, including the formation of acetaminophen-glucuronide and acetaminophen-sulfate, showed higher levels in the digestive conditions including chyme than it did under similar conditions without chyme. This result was similar to the in vivo result obtained in the rat study, which demonstrated the apoptosis of liver cells caused by acetaminophen toxicity. In the following study [31], the μCCA was applied to assess the effect of the oral absorption of nanomaterials after first pass metabolism (Figure 2A). The GI μCCA and multi chamber μCCA (representing the body, including the liver) were used, in which Caco-2/HT29-MTX and HepG2 cells were cultured. Carboxylated polystyrene nanoparticles were used as a representative nanomaterial. The results revealed that the presence of the Caco-2/HT29-MTX barrier led to the partial absorption of nanoparticles. It was observed that a fraction of the nanoparticles were transferred to the liver part and affected the HepG2 cells. Liver cell damage was demonstrated based on the detection of aspartate aminotransferase (AST). In this system, the damage caused by the mixed effect of the interaction between organs was more generalized than the damage observed in the single tissue system.

Additionally, the co-culture of HepG2 cells and other parenchymal cells (liver sinusoidal endothelial cells, hepatic stellate cells, and Kupffer cells) has been reported to improve the cell growth and function of the liver through crosstalk between them. Several studies have applied the co-culture of liver-specific cells to mimic the liver microenvironment. Based on these results, Shuler and coworkers recently developed a modular system for studying first pass metabolism [39] (Figure 2B). The system allows for a plug-and-play approach. Caco-2 cells were cultured in the chamber of the GI tract chip. Primary human hepatocytes and non-parenchymal cells were cultured in the chamber of the liver chip. After the GI tract and liver tissues were mature, each chip was assembled and maintained on the rocking platform. Through rocking movements, the cell culture medium was circulated between the GI tract chamber and the liver chamber. After 14 days of co-culture, the GI tract maintained their viability with barrier function. The liver cells showed low rates of liver cell death with comparable metabolic function, which is comparable to the results of the single liver organ chip system. These studies described that the co-culture of organ-specific cells are advantageous for the simulation of first pass metabolism. Although the superiority of the co-culture of organ-specific cells was described in a single organ chip system, such as a gut-on-a chip system and a liver-on-a chip system, most systems simulating first pass metabolism are still only incorporating Caco-2 and HepG2 cells. Therefore, the co-culture of organ-specific cells for a better prediction of first pass metabolism-based drug metabolism is encouraged.

### 3.3. Primary Cells and Tissue Slices

**Figure 3:**
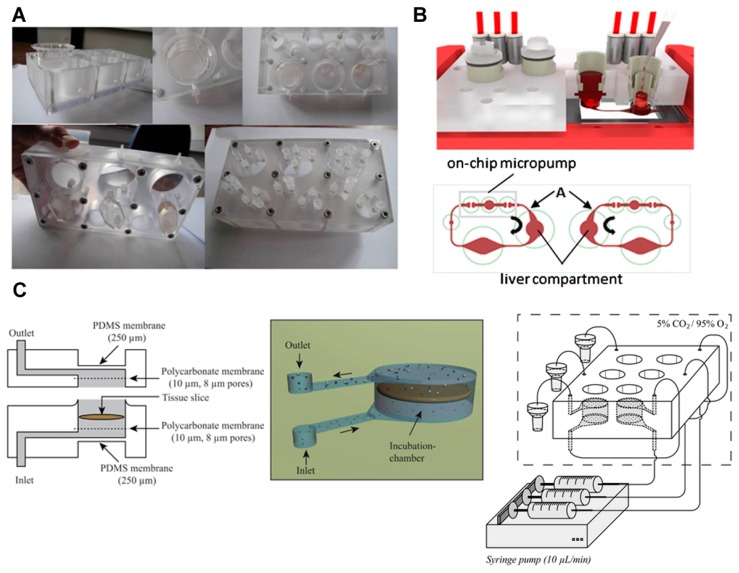
First pass metabolism models based on primary cells and tissue slices. (**A**) Integrated Insert in Dynamic Microfluidic Platform (IIDMP) for the simulation of first pass intestinal and liver metabolism. In the insert well, Caco-2 cells were cultured. In the liver compartment, HepG2/C3A, freshly isolated rat primary hepatocytes, and human cryopreserved hepatocytes were cultured. Image reprinted from [40] with the permission of John Wiley & Sons. (**B**) Schematic illustration of the multi-organ chip (MOC) for the co-culture of liver and intestinal cells. Image reprinted from [41] with the permission of Elsevier. In the intestine compartment, human primary intestinal epithelial cells were cultured. In the liver compartment, the human HepaRG cell line was cultured with human primary hepatic stellate cells. (**C**) Microfluidic system for the culture of precision-cut intestinal and liver slices. Image reprinted from [30] with the permission of The Royal Society of Chemistry.

As many researchers have reported that primary cells are better for simulating and predicting the metabolic function of organs than tumor derived cell lines, primary cells were incorporated into the organ-on-a chip system. Leclerc and coworkers [40] used a previously developed bioreactor that consists of transwell polycarbonate inserts and microfluidic biochips to investigate the first pass metabolism of acetaminophen in the intestine and liver (Figure 3A). For this, the Caco-2 cells cultured in an insert well were connected to a liver compartment on a microfluidic chip. In the liver compartment, HepG2/C3A, freshly isolated rat primary hepatocytes, and human cryopreserved hepatocytes were cultured for comparative analysis. The acetaminophen was transported through the intestinal barrier and acetaminophen sulfate was produced by the synergistic metabolism of the intestine and liver. Acetaminophen glucuronide was produced with the addition of rat primary hepatocytes and human hepatocytes. By using a PK model, in vitro intrinsic liver and intestinal clearances and the kinetic parameters for the permeability of acetaminophen and acetaminophen sulfate were estimated. The findings allowed for the prediction of in vivo hepatic and intestinal clearances based on a drug’s bioavailability. This study demonstrates that in silico/in vitro methods using microfluidic chips can be applied to predict and evaluate organ-to-organ interactions, as well as drug absorption, distribution, and metabolism.

Marx and coworkers developed a multi-organ-chip (MOC) system. The system consisted of a microchannel, a chamber for culture, and an on-chip peristaltic micropump. The PDMS-membrane-based peristaltic pump was operated by pressure and its operation enabled the flow of culture medium. As an example, MOC was applied to the liver-intestinal barrier model for homeostatic long-term co-culture (Figure 3B) [41]. Human primary intestinal epithelial cells cultured using this system showed a columnar epithelial cell morphology. Most proliferating cells were located at the lowest layer in the system. The TEER value was kept constant and close to the physiological TEER values. The expression of MRP-2, the active transporter pump, and cytokeratin 8/18 was observed using this system. These liver-intestinal models were used to simulate the effects of repeated dose administration of a model substance (troglitazone) through the oral routes, and the response was demonstrated by measuring the mRNA levels and immunohistochemical analyses.

To construct a more organ relevant model, organ tissues have been integrated into microfluidic systems. One example is the use of precision-cut tissue slices extracted from rats through a surgical procedure. Because precision-cut tissue slices retain a complex tissue structure and include all cell types, it can simulate the in vivo microenvironment and organ-specific metabolism. Van Midwoud et al. [30] developed a system to study the interaction between organs, using the intestine and liver slices of a rat (Figure 3C). The system consisted of an inlet, outlet, and a tissue chamber. The tissue slices were incorporated between polycarbonate membranes in the chamber. The membranes enabled the distribution of medium around the slices, and the slices could be suspended in the medium. The thickness of the PDMS membrane in the chamber was thinner than that in other parts, which allowed for the supplement of oxygen and carbon dioxide to the medium. After incorporating the intestine and liver slices into the system, the intestine and liver chambers were connected by tubing. The metabolites generated from the intestinal slices were transported to the liver slices to simulate the first pass metabolism, while the interactions between both organs were simulated based on the homeostasis of bile acid. The exposure of the two organs to the primary bile acid induced the expression of fibroblast growth factor 15 in the intestinal slice, which down-regulated cytochrome P450 (CYP) 7A1 expression in the liver slice. The downregulation of CYP7A1 in both organ systems was higher than that in the liver system alone, verifying the hypothesis.

Although it is difficult to maintain the function and viability of primary cells and precision-cut-slices, they are advantageous for mimicking organ functions more relevant to in vivo systems. Therefore, their incorporation into the system should be considered when multi-organ systems are constructed.

## 4. Multi-Organ Model with More than Three Organs

**Figure 4:**
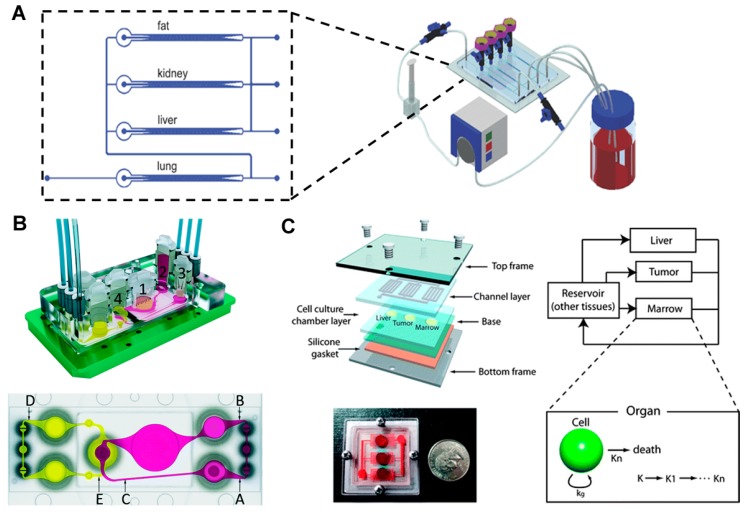
Multi-organ model (**A**) Multi-channel 3D microfluidic cell culture system for drug screening. Image reprinted from [44] with the permission of The Royal Society of Chemistry. (**B**) Microfluidic four-organ chip for mimicking the ADME processes. Image reprinted from [47] with the permission of The Royal Society of Chemistry. (**C**) Gravity-induced flow-based microscale cell culture analog (μCCA). A PK-PD model and a μCCA were combined to study the mechanism of action of the drug. Image reprinted from [48] with permission of The Royal Society of Chemistry.

Except two organ systems for simulating first pass metabolism, several organ-on-a chip systems have been developed to simulate interactions between various organs, and various types of cells have been co-cultured on a single device [42,43,44]. Zhang et al. [44] developed a multi-channel 3D-microfluidic cell culture system (3D μFCCS) with four different cells (A549, C3A, HK-2, and HPA cells), separately cultured to simulate a multiple organ system (the lungs, liver, kidney, and lipid tissues) (Figure 4A). Each cell type was fixed between the micropillar arrangements, while the medium was perfused to the center from the two-way channels by diffusion. The medium was perfused to the other channels after it perfused through the lung parts. The limited interaction between the different cell types was verified by mixing the cells with gelatin microspheres. The controlled release of transforming growth factor β1 enabled the maintenance of the functions of each cell type, including the albumin secretion of C3A cells, PROD enzymatic activation of A549 cells, γ-glutamyltransferase enzymatic activation of HK2 cells, and adiponectin secretion of HPA cells.

Marx and coworkers [45] applied their MOC system for the co-culture of liver and skin biopsies. The co-culture of skin biopsy samples and the spheroid of HepRG liver or primary hepatic stellate (HHSteC) cells on the chip verified the system’s suitability for representing various tissues. For co-cultures that lasted for 14 days, the liver and skin tissue were exposed directly to fluid flow, and crosstalk between tissues was observed, indicating a balance in the albumin production by the liver and its consumption by the skin. Upon exposure to a toxic substance, troglitazone, for six days, the liver tissue showed a dose-dependent sensitivity, which was determined based on the levels of proteins and the mRNA expression of enzymes (P450 3A4). A stable long-term (28 days) performance was demonstrated through co-cultures performed on a transwell insert-based system. In this system, the nutrient supply was less efficient than that in the system in which cells were cultured without the transwell inserts; however, the tissue was exposed to the air-liquid interface and was shielded from the shear stress caused by the fluid. This allowed epidermal cell differentiation. Based on the expression of enzymes, the metabolic activity of the liver tissues and epidermis was verified after the 28-day culture. Furthermore, this group tried to simulate the transport function of human vasculature on a chip [46]. The system was covered with blood vessel cells, primary human dermal microvascular endothelial cells (HDMECs). After four days of cultivation, stable microvascular circuits were demonstrated through cellular viability and vascular functionality. By using a peristaltic on-chip micropump, pulsatile shear stress was applied to the cells in a physiological range. The shear stress induced changes in cell physiology, verified by cell alignment, cellular morphology, and the distribution of filamentous actin. In addition, the reduction of vessel branching and vessel diameter in the organ compartments was observed by applying a two-photon laser ablation technique to the inserts. The HDMECs were distributed in all branched microchannels. Based on these results, the vascularized system was added to the liver and skin biopsies co-culture system [41]. Although the interactions between endothelial cells and liver spheroids or skin biopsies were limited, the overall metabolic activities were stable based on the glucose consumption and lactate production. The skin biopsies verified the expression of cytokeratin 10 and 15. The liver-vasculature-skin biopsy model was used to simulate the effects of repeated dose administration of troglitazone through the systemic routes, and the MOC platform was expanded to simulate ADME (Figure 4B) [47]. Similarly, four organ chips were manufactured for culturing the intestine, liver, skin, and kidney tissues. Impressively, the functions and viability of the four organs was maintained over 28 days in co-culture. The functional integrity of the small intestine and kidney proximal tubule cell barriers was verified based on the TEER values of the intestinal tissues, constant LDH levels, and stable glucose balance between the intestinal lumen, the surrogate blood circuit, and the excretory circuit of the system. The human small intestine epithelia exhibited perfect physiological polarization and formed stable 3D villi-like structures of up to 270 μm in height. The 3-D spheroids of hepatocytes preserved the initial well-defined 3-D architecture over the 28 days of culture. Skin biopsies maintained in vivo-like architecture at a physiological air–liquid interface, such as a stratified stratum corneum. The sodium-potassium pump in the human proximal tubule epithelia maintained its transport function. The metabolism and gene analysis verified that all four tissues showed homeostasis, physiological functions, and metabolic capabilities for at least 28 days. This study successfully demonstrated that multiple organ co-cultures could be successfully maintained on the chip.

## 5. PK-PD Modeling-Based Multiple Organ Model

As noted earlier, the combination of PK-PD models and a microfluidic co-culture system could work as a novel platform for reproducing the action of drugs in the body. Several initial attempts have been made and reported, some of which have been already mentioned. Here we describe a few other studies that have specifically focused on creating a physical realization of a PK-PD model using a microfluidic co-culture system. The Shuler group [49] described a PK-PD model-based μCCA device, which allowed for the evaluation of drug metabolism and prediction of the inter-organ interactions between the liver and other organs in the human body. Subsequently, the design, fabrication, and operation of a three-chamber microscale cell culture analog (μCCA) system were attempted; the chambers represented the lung, liver, and other tissues. The system consisted of a silicon substrate that contained fluidic networks and chambers, confined within Plexiglass housing. Mammalian cells (L2 and H4IIE) were cultured for over 24 h and the cell viability was then examined. The oxygen exchange rate for cell cultures was measured using an integrated fluorescent-based oxygen sensor [50]. The μCCA system was validated using naphthalene as a model toxic chemical [51]. The system contained four compartments (representing the lung, liver, other tissues, and fat). The lung and liver cells were cultured in two separate chambers. The tissue and fat compartments did not contain cells, but had a controlled fluid flow that was induced by channel geometry to mimic in vivo residence times. This was intended so that the drug distribution in the chip would mimic that of the human body. After the medium passed through the inlet, the fluid moved through the lung compartment and was distributed to the liver, fat, and other tissue compartments. The fluid was then combined and recirculated from the outlet to the inlet for recirculation. Naphthalene, added to the circulating culture medium, was metabolized and generated reactive metabolites (Naphthoquinone and naphthalene diol) by a P450 enzyme in the liver compartment; the metabolite circulated and induced GSH depletion in the lung and liver cells (L2 and C3A cells, respectively), resulting in cell death. L2 cells were more sensitive to GSH depletion, as the GSH re-synthesis rate of C3A cells was faster than that of L2 cells. Cell cultures performed in a 2D monolayer failed to replicate the physiological response of native tissue in vivo, mainly because 2D monolayer culture systems cannot imitate cell-to-cell and cell-to-ECM interactions, which are important for maintaining the physiological function of cells. Therefore, a 3D hydrogel-based cell culture system is required for tissue-specific functions and differentiated states of cells [52]. Colon cancer cells (HCT-116), hepatoma cells (HepG2/C3A), and myeloblasts (Kasumi-1) were embedded and cultured in the tumor, liver, and marrow chamber, respectively. The chambers were connected by the microfluidic network, mimicking the blood flow around the target organs. The cytotoxic effect of Tegafur, an oral prodrug of 5-fluorouracil (5-FU), was compared in a 96-well microtiter plate and the µCCA. The hypothesis set up by the authors was that the presence of liver metabolism in the µCCA would produce the active drug, which would kill tumor cells. As predicted, in the µCCA, Tegafur was converted to 5-FU by the P450 enzyme in the liver, and consequently induced the death of cancer cells. The μCCA system was used to mimic in vivo pharmacokinetic and pharmacodynamic drug profiles. In addition, a gravity-induced fluidic system was developed and the toxicity of 5-FU was analyzed by using a PK-PD model (Figure 4C) [48,53]. Cells showed a higher sensitivity to 5-FU using this system compared to the results obtained using the static system. In addition, the difference in responses based on cell types was distinct using the dynamic system compared to responses generated using the static system. A corresponding PK-PD model was constructed to quantify the action of the drug in the chip, providing insight into the in vivo drug mechanism.

## 6. Remaining Challenges and Conclusions

Several challenges remain in the organ-on-a-chip-based field, which include the issue of cell culture medium and the correct down scaling of the chip to represent the human body. Various solutions have been considered and proposed. First, different cells use different culture media, as each cell type requires a different medium composition. The development of a universal, common medium that would support the culture of all cell types is needed to operate MOC. Secondly, the MOC should be correctly designed, based on the relative size of each organ and the flow rate to reflect their physiological state inside the body. If the relative size of each organ and the flow rate are not balanced, the effect of the drug on the cellular activity could easily be distorted. The simulation of in vivo metabolism can only be achieved with the correct correlation between relative size and the metabolic rate. Several approaches, including traditional allometric scaling and the method based on matching residence times have been suggested to solve this scaling issue [54]. Allometric scaling includes the consideration of the body size and the metabolic rate between each organ. Therefore, the scaling variable can be used to design various types of MOC [55]. When the chip is designed, the residence time of the blood inside the body should be considered. The volume and mass of the organ and blood compartments inside the chip can be changed, depending on the variables. The consideration of retention time allowed for MOC-based drug screening because the retention time of drugs that occurs in the human body could be simulated by MOC. Furthermore, metabolism-based functional scaling could provide information on the function and reaction of drugs inside the human body [54]. Currently, no universal solution for the issue of scaling has been accepted. It is possible that the optimal method also depends on the specific purpose of an individual MOC. Thirdly, an organ-specific environment should be reproduced in the multi-organ system. In a single organ chip system, various components of an organ-specific environment have been considered for better cell viability with the comparable metabolic function when compared with that of the in vivo system. For example, the 3D structure of villi and peristaltic movement was realized by microfabrication technologies for mimicking the gut system [56,57]. For mimicking the liver system, a co-culture of hepatocyte and non-parenchymal cells with a 3D hydrogel-based environment has shown long-term culture with the metabolic function [58,59,60]. However, some multi-organ systems do not reflect this type of achievement. A similar environment with an in vivo system would provide a more precise prediction for the ADME of drugs. Lastly, we summarized the incorporation of primary cells and tissue slices so that the multi-organ system mimics the in vivo system more closely. However, it is very difficult to maintain their function. Human-induced pluripotent stem cells (iPSCs) can be proposed as an alternative [61]. The iPSCs can allow for the construction of more relevant systems compared with the human body and can provide a personalized model through an organoid in the chip system.

In this review, we highlighted the current reported research into microtechnology-based systems that address multi-organ interactions. First, we introduced the concepts of PK-PD modeling. In addition, we introduced several gut- and liver-based microfluidic systems for reproducing the first pass metabolism. To study the dynamic interactions between various organs, the concept of a multiple organ model was introduced. The results enabled the more accurate prediction of drug efficacy and toxicity based on multiple-organ interactions, which can be complemented by PK-PD modeling. However, these studies were still limited to simple metabolic reactions and many issues still need to be overcome. However, the development of improved systems will provide a foundation for the development of a human body chip system, which in turn, would enable the accurate prediction of general drug reactions. Furthermore, the studies highlighted in this review have the potential to support animal- and human-based researches for drug screening, the development of drugs, and the study of disease mechanisms.
